# Job satisfaction, depression severity and quality of life ratings of perioperative nurses in robotic-assisted and laparoscopic surgery

**DOI:** 10.1007/s11701-023-01764-y

**Published:** 2024-01-13

**Authors:** Dilara Nur Turgut, Ece Tuncel, Aslihan Palta, Mehtap Tektas, Melih Balci, Ozer Guzel, Tanju Keten, Yilmaz Aslan, Altug Tuncel

**Affiliations:** 1 University of Health Sciences School of Medicine, Ankara City Hospital Section for Minimally Invasive and Robotic Surgery, 06800 Ankara, Turkey; 2 George Herbert Walker School of Business and Technology, Department of Management, Webster University, St. Louis, MO 63119 USA; 3 Department of Urology, Section for Minimally Invasive and Robotic Surgery, University of Health Sciences School of Medicine, Ankara City Hospital, Oncology Building, Ground Flour/C Block, Room: 60031500, 06800 Ankara, Turkey

**Keywords:** Perioperative nurses, Job satisfaction, Depression, Quality of life

## Abstract

The rapid introduction of technological developments into healthcare systems adds another layer of complexity to the already demanding jobs of nurses, particularly for those working in perioperative care. In the present study, our primary aim is job satisfaction, whereas the secondary outcomes are psychological well-being and quality of life (QoL) ratings of perioperative nurses who take part in robotic-assisted and pure laparoscopic surgery. A total of 101 perioperative nurses in six different centers were included in the study. Fifty-one of the nurses were working in robotic-assisted laparoscopic surgery and 50 of them were working in pure laparoscopic surgery. All participants responded to Minnesota Job Satisfaction Questionnaire (MJSQ), Beck Depression Inventory (BDI) and SF-36 QoL Measurement Survey. The two groups did not differ in their total MJSQ, BDI and SF-36 QoL scores (*p*_MJSQ_:0.066, *p*_BDI_:0.329, *p*_SF-36-QoL_:0.136). In addition, there were no differences between the two groups in their intrinsic job satisfaction and extrinsic job satisfaction sub-scores (*p*_intrinsic_: 0.473, *p*_extrinsic_:0.121). Overall, 18.9% of the nurses reported having moderate to extreme depressive symptoms and most of them (87.1%) had low to moderate levels of job satisfaction. Finally, QoL ratings was generally at moderate levels. Perioperative nurses who work in robotic-assisted laparoscopic surgery do not differ from those working in pure laparoscopic surgery in terms of their job satisfaction, psychological well-being, and QoL ratings. In addition, across groups’ psychological well-being, job satisfaction, and QoL ratings were not particularly high, suggesting that more attention needs to be paid to improving the work conditions of perioperative nurses.

## Introduction

Among health professionals, nurses constitute the largest workforce. Thus, it is vital to increase the quality of service that nurses provide for positive patient outcomes [1]. However, nursing is generally accepted as a high-risk profession in terms of burnout and work-related stress, with nurses in certain specialties experiencing particularly high levels of stress [2, 3]. Hospital-employed nurses have higher rates of mental health challenges than the general population [4]. Particularly, depression, anxiety, and stress are rated high, reducing nurses’ quality of life (QoL) ratings [5]. As nurses work in interdependent settings, these mental health and QoL concerns could have serious implications for patients, other healthcare professionals, and healthcare organizations at large.

In addition, the rapid introduction of technological developments in healthcare systems adds another layer of complexity to the already demanding jobs of nurses, particularly for those working in perioperative care. Robotic-assisted laparoscopic surgery has changed the physical and interpersonal context of surgical teams compared to pure laparoscopic surgery, potentially impacting nurses’ job satisfaction as well as subsequent patient outcomes. Robotic-assisted and pure laparoscopic surgery nursing differ from each other in some aspects. Robotic-assisted surgery nurses are responsible for preparing the robotic surgical system and controlling it during surgery. These nurses have the knowledge of sterile and non-sterile parts of the robot. Their responsibilities include checking the patient’s position before and during surgery, placing surgical instruments on robotic arms, applying relevant procedures in an emergency situation, monitoring and interpreting the information in the system to keep the patient safe [6, 7]. Finally, they keep surgical materials available for the possibility of conversion to laparoscopic or open surgery. Due to the complexities introduced by new technology, robotic surgery nurses perform varied, specialized tasks that laparoscopic surgery nurses do not perform. Yet, despite the changing landscape of work and increased responsibilities, there is scarcity of research examining the effects of new technologies on nurses’ job satisfaction.

Job satisfaction refers to a person’s attitudes toward work, including their emotional states when they reach their work-related goals expectations in work life [8]. Satisfaction with the work environment has implications for employees’ relationships as well as their own psychological well-being.

The main objective of this study is to compare the job satisfaction of nurses in robotic-assisted laparoscopic and pure laparoscopic surgery. We also examine whether two groups of nurses differ in terms of their psychological well-being (i.e., depression) and QoL ratings.

## Materials and methods

This study was approved by the Institutional Ethics Committee (Approval #: E1-20-356) and performed in accordance with the ethical standards stated in the 1964 Declaration of Helsinki. Informed consent was obtained from all participants. This cross-sectional study was based on a paper–pencil survey conducted from June 2020 through September 2020.

A total of 101 perioperative nurses who had been working in robotic-assisted laparoscopic (*n*: 51; 41 female, 10 male) and pure laparoscopic (*n*: 50; 40 female, 10 male) surgery in six different centers (3 government and 3 private hospitals) were included. Participants were licensed registered nurses with at least 1 year of employment at their current institution.

### Measures

Our primary outcome is job satisfaction, whereas the secondary outcomes are psychological well-being and quality of life ratings. Accordingly, participants filled out the Minnesota Satisfaction Questionnaire (MSQ), Beck Depression Inventory (BDI), and SF-36 QoL Survey.

The short version of the MSQ was used to measure job satisfaction among nurses. This questionnaire is a 20-item self-report measure that examines two aspects of job satisfaction: (1) *intrinsic satisfaction* (i.e., how employees feel about the nature of their job tasks), (2) *extrinsic satisfaction* (i.e., how employees feel about aspects of the work situation that are external to the job tasks such as work conditions). Each sub-scale consists of ten items scored on a five-point scale ranging from 1 (*very dissatisfied*) to 5 (*very satisfied*). The total score obtained from adding intrinsic and extrinsic satisfaction sub-scores indicates overall job satisfaction. The overall score ranges from 20 to 100 such that scores ranging from 20 to 47 indicate low job satisfaction, 48–76 indicate moderate job satisfaction, and 77–100 indicate high level of job satisfaction [9].

The BDI is a 21-item scale measuring various symptoms of depression. It has 21 items addressing somatic and affective aspects of depression. Each item consists of four alternative responses rated from 0 to 3 according to the severity of the symptom (*0* = *non-existent; 3* = *severe*). Participants were asked to choose the response closest to their state during the past week. Participants’ responses to 21 items are added to compose a depression score, with higher scores indicating higher levels of depression. Individual scores were from 0 to 66. Scores 1–10 indicate no depression, 11–16 indicate mild mood disturbance, 17–20 indicate borderline clinical depression, 21–30 indicate moderate depression, 31–40 indicate severe depression,and scores over 40 indicate extreme depression [10].

The SF-36 QoL comprises 36 questions covering eight aspects of health status: physical functioning, role-physical (role limitations due to physical health problems), bodily pain, general health, vitality, social functioning, role-emotional (role limitations due to emotional problems), and mental health. The scores of questions relating to each scale were summed and rescaled to a 100-point scale, where 100 is the best possible score and 0 the worst possible score [11].

A paper–pencil survey was distributed to the participants by three of the authors (D.N.T., A.P. and M.T.) at National Urology Nurses Society’s Annual Meeting in 2020 as well as National Surgery and Perioperative Nurses Society’s Annual Meeting in 2020.

## Statıstıcal analysıs

Statistical analysis was performed using the Statistical Package for the Social Sciences (SPSS, Chicago, IL) version 28.0.1. Descriptive statistical data for continuous variables were expressed as mean and standard deviation. *T* tests were conducted to compare the two groups in their job satisfaction, psychological well-being, and quality of life scores. A *p* value of less than 0.05 was considered significant when testing the differences between the nurses in their ratings.

## Results

The mean age of the participants was 34.8 (23–51) years. 80.2% of the participants were female and 19.8% were male. Moreover, most participants (88.1%) had a bachelor’s degree. The majority of the participants (62.4%) had more than 10 years of work experience. There were no differences between perioperative nurses who had been in robotic-assisted laparoscopic and pure laparoscopic surgery regarding their demographic parameters. The demographic data of the participants are summarized in Table 1.

**Table 1:** The demographic data of the participants

Parameters	Robotic-assisted laparoscopic surgery group (*n* = 51)	Pure laparoscopic surgery group (*n* = 50)	p*
Age (yr) (Mean±SD)	35.1**±**7.5	34.6**±**6.4	0.742
Gender (n)			0.961
Female	41	40	
Male	10	10	
Education level (n)			0.552
High school	4	8	
University	47	42	
Working duration (n)			0.205
1–4 year	11	6	
5–10 year	9	12	
> 10 year	31	32	
Marital status (n)			0.904
Single	21	20	
Married	30	30	

*SD* standard deviation

^*^Student’s *T* test

We first examined the effects on our primary dependent variable, namely job satisfaction. The results indicated that 21.8% of nurses had low levels of job satisfaction, 65.3% had moderate levels of job satisfaction, and 12.9% had high levels of job satisfaction. We did not find significant differences between the groups in terms of their total MSQ score (*p*: 0.066). In addition, intrinsic and extrinsic job satisfaction sub-scores of MSQ were not significantly different between the groups (*p*_intrinsic_: 0.473, *p*_extrinsic_: 0.121).

Then, we examined the effects on our secondary dependent variables, namely BDI and quality of life. BDI scores indicated that 39.6% of nurses had no depressive symptoms, 31.7% had mild mood disturbance, 9.9% had borderline clinical depression, 13.9% had moderate depression, and 5% had severe or extreme depression. There were no significant differences between the groups in their BDI scores (*p*: 0.329). Finally, for SF-3 6 QoL, mean physical functioning ratings appear to be on the higher end of the scale (mean = 82.13, *SD* = 17.99). Participant ratings were generally at moderate levels on other aspects such as energy–vitality, mental health, and role limitations related to emotional problems. Finally, two groups of nurses did not significantly differ in terms of their QoL ratings (p:0.136). The results are shown in Table 2.

**Table 2:** Groups means for variables

Parameters	Robotic-assisted laparoscopic surgery group (*n* = 51)	Pure laparoscopic surgery group (*n* = 50)	p*
MSQ			
Total	62.06±14.97	56.52±14.97	0.066
Intrensic satisfaction sub-score	38.80±9.57	37.31±11.15	0.473
Extrinsic satisfaction sub-score	22.65±7.12	20.25±8.23	0.121
BDI	14.80±8.89	13.02±9.40	0.329
SF-36 QoL			
Physical functioning	81.08±17.21	83.20±18.87	0.556
Physical limitation	49.51±34.82	51.50±36.57	0.780
Emotional limitation	51.63±37.90	49.99±32.48	0.816
Energy–vitality	48.14±20.40	51.50±18.61	0.389
Mental health	56.16±18.37	58.96±18.90	0.451
Social functioning	64.95±20.16	64.00±21.38	0.819
Pain	61.52±22.82	69.65±22.85	0.077
General health	56.37±16.10	61.50±18.11	0.136

The scores are presented as mean ± standard deviation

*MSQ* Minnesota Satisfaction Questionnaire, *BDI* Beck Depression Inventory, *QoL* quality of life

^*^Student’s *T* test

## Discussion

Nurses who are overworked likely experience negative emotional states and adverse health effects due to burnout that in turn may reduce their performance and quality of care [1, 12, 13]. In addition, adoption of new technologies in the operating room could be motivating for perioperative nurses, yet may place additional job demands that could be difficult to manage. Perioperative nurses have varied responsibilities, including ensuring that they are correctly ‘scrubbed up’, preparing the instruments, trolleys and sterile supplies needed for the surgery, maintaining a sterile environment, preparing the patient, providing skilled assistance to the surgeon during the operation, and performing the swab/instrument count at the end of the procedure [14]. In addition to providing quality patient care, operating room nurses should be an effective team member who could work with multiple healthcare professionals [15].

Intense workload of operating room nurses likely increases stress, burnout, and anxiety, and decreases their job satisfaction. For example, a study by Boyle et al. investigated the job satisfaction of 55,516 registered nurses in 206 hospitals in the USA [16]. They found that job satisfaction varied by work unit and that perioperative nurses were least satisfied with their jobs due to the unique demands of their work environment. Given these considerations, it is essential to examine the well-being and job satisfaction of perioperative nurses and understand whether and how the adoption of new technologies influences not only their job demands, but also their psychological outcomes [13]. Ultimately, the quality of patient care rests on the well-being and job satisfaction of nurses in the operating room [12].

The current study revealed that perioperative nurses in general were moderately satisfied with their jobs. Between the robotic-assisted and pure laparoscopic surgery nurses, overall job satisfaction scores did not significantly differ. We also did not find significant differences in the intrinsic and extrinsic job satisfaction scores of two groups which requires further attention considering recent research findings. Intrinsic job satisfaction refers to the satisfaction gained from actual job tasks [17, 18]. For example, finding meaning in one’s contributions or feeling a sense of achievement due to being a part of new initiatives are sources of intrinsic job satisfaction. A recent review of qualitative research studies on the experiences of robotic-assisted laparoscopic surgery nurses revealed that these nurses expressed a positive attitude toward incorporating the latest surgical innovations into their daily practices and that they were proud to be part of a team that employs this latest technology [19]. Accordingly, it could be expected that perioperative nurses who work in robotic-assisted laparoscopic surgery would experience higher intrinsic job satisfaction than those who work in pure laparoscopic surgery due to the novelty and usefulness of this new technology. However, we did not find such a difference in our data.

In addition, perioperative nurses working in robotic-assisted laparoscopic surgery also voiced concerns related to increasing importance of teamwork, shifting job demands, changes in workload, and intense training requirements [19, 20]. These factors relate to extrinsic job satisfaction, which involves satisfaction related to external factors such as working conditions, relationships with co-workers and salary [17, 18, 21]. While nurses working in robotic-assisted laparoscopic surgery could be expected to experience lower extrinsic job satisfaction than those working in pure laparoscopic surgery due to the challenges related to working with new technology, we did not find a difference between the groups. We should note that two groups of nurses are not paid differently in our context, partially accounting for the lack of difference in their extrinsic satisfaction. We call for future research to further examine how new technologies affect perioperative nurses’ job satisfaction, especially when new job demands result in increased pay.

Nurses spend most of their working time interacting directly with patients and/or their relatives. In addition, nurses often witness tragic instances, including illness, trauma, and even death, which could be physically demanding and psychologically stressful. Negative psychosocial factors in the working environment can adversely affect the psychological and physical well-being of nurses [22]. Welsh found that 35% of surgical hospital nurses scored above the cutoff for mild to moderate depressive symptoms [23]. In our study, 18.9% of the nurses reported having moderate to extreme depressive symptoms. We did not find a statistically significant difference in terms of BDI scores between two groups of nurses. This finding indicates that the type of surgical environment does not relate to nurses’ mood states.

QoL is expressed in terms of an individual’s sense of satisfaction, which consists of factors such as work quality, satisfaction with personal life, and having financial independence. Welsh reported that work attributes including appropriate supervision, cooperation, and relationships with patients play a role in the QoL ratings of nurses [23]. A study by Orszulak et al. found the QoL level of nurses to be around the mid-point of the scale [24]. The nurses in their study reported the best QoL rating in the psychological domain and the worst in the physical domain. In our study, QoL ratings were also at moderate levels. However, QoL ratings in the physical domain were higher than those in other domains. We again did not find significant differences between two groups of nurses in their QoL ratings. This result suggests that the type of surgical environment does not relate to the QoL perceptions of perioperative nurses.

Perioperative nurses in our sample work in either public or private hospitals. We should note that our healthcare system is highly standardized, with minimal differences in working hours, work conditions and expectations for perioperative nurses working in public and private hospitals. Given these similarities, we do not expect the work setting to impact the variables of interest in this study (i.e., MSQ, BDI, and SF-36 QoL). In addition, Kaushik et al. revealed that the prevalence of depression, anxiety and ratings of work stressors were comparable for nurses working in public and private settings [25]. Based on these findings, we do not expect the work context to influence on our results.

To our knowledge, this study is one of the first studies to compare job satisfaction, psychological well-being and QoL perceptions of nurses who work in robotic-assisted and pure laparoscopic surgery. We found that there were no differences between the groups in terms of these variables. Generally, these findings indicate that, regardless of the workload and work context, attention should be paid to enhancing the well-being of nurses to enhance effectiveness of patient care.

A few limitations of this study must be noted. First, this study includes a narrow group of nurses in our national healthcare system, including nurses from six hospitals (three public and three private) in different regions. While we do not have a specific reason to expect different results depending on the region and type of hospital, caution is needed in generalizing the results to other settings. Second, the cross-sectional nature of the study should be considered when interpreting the results.

## Conclusion

Our results show that job satisfaction, psychological well-being and QoL ratings were similar between perioperative nurses who work in robotic-assisted and pure laparoscopic surgery. In our sample, 18.9% of the nurses reported having moderate to extreme depressive symptoms and most of them (87.1%) had low to moderate levels of job satisfaction. Finally, QoL ratings were generally at moderate levels. While the QoL and psychological well-being ratings could be impacted by factors outside of work, healthcare systems should focus on increasing nurse satisfaction to improve the quality of patient care.

## Data Availability

The data that support the findings of this study are available from the corresponding author, upon reasonable request.
